# Higher‐Order Topological States in Surface‐Wave Photonic Crystals

**DOI:** 10.1002/advs.201902724

**Published:** 2020-01-27

**Authors:** Li Zhang, Yihao Yang, Zhi‐Kang Lin, Pengfei Qin, Qiaolu Chen, Fei Gao, Erping Li, Jian‐Hua Jiang, Baile Zhang, Hongsheng Chen

**Affiliations:** ^1^ Interdisciplinary Center for Quantum Information State Key Laboratory of Modern Optical Instrumentation College of Information Science and Electronic Engineering Zhejiang University Hangzhou 310027 China; ^2^ ZJU‐Hangzhou Global Science and Technology Innovation Center Key Laboratory of Advanced Micro/Nano Electronic Devices & Smart Systems of Zhejiang The Electromagnetics Academy at Zhejiang University Zhejiang University Hangzhou 310027 China; ^3^ Division of Physics and Applied Physics School of Physical and Mathematical Sciences Nanyang Technological University 21 Nanyang Link Singapore 637371 Singapore; ^4^ Centre for Disruptive Photonic Technologies The Photonics Institute Nanyang Technological University 50 Nanyang Avenue Singapore 639798 Singapore; ^5^ School of Physical Science and Technology, and Collaborative Innovation Center of Suzhou Nano Science and Technology Soochow University 1 Shizi Street Suzhou 215006 China

**Keywords:** higher order photonic topological insulators, photonic crystals, topological photonics

## Abstract

Photonic topological states have revolutionized the understanding of the propagation and scattering of light. The recent discovery of higher‐order photonic topological insulators opens an emergent horizon for 0D topological corner states. However, the previous realizations of higher‐order topological insulators in electromagnetic‐wave systems suffer from either a limited operational frequency range due to the lumped components involved or a bulky structure with a large footprint, which are unfavorable for achieving compact photonic devices. To overcome these limitations, a planar surface‐wave photonic crystal realization of 2D higher‐order topological insulators is hereby demonstrated experimentally. The surface‐wave photonic crystals exhibit a very large bulk bandgap (a bandwidth of 28%) due to multiple Bragg scatterings and host 1D gapped edge states described by massive Dirac equations. The topology of those higher‐dimensional photonic bands leads to the emergence of in‐gap 0D corner states, which provide a route toward robust cavity modes for scalable compact photonic devices.

## Graphical abstract

Topological corner states are experimentally demonstrated in planar surface‐wave photonic crystals, which have advantages such as large bandgaps and small lattice constants. Such corner states arise from the topology of the gapped edge states that live in the bulk bandgap of photonic crystal. This work provides a pathway toward robust cavity modes for scalable compact electromagnetic‐wave/photonic devices.

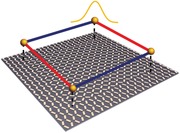

Photonic topological insulators (PTIs)^1^, ^2^, ^3^, ^4^, ^5^, ^6^, ^7^, ^8^, ^9^, ^10^, ^11^, ^12^, ^13^, ^14^, ^15^, ^16^, ^17^, ^18^, ^19^ host unprecedented edge states such as chiral or helical edge states in 2D PTIs^4^, ^9^, ^10^, ^12^, ^13^, ^18^ and Dirac‐fermion‐like surface states in 3D PTIs.^19^, ^20^ The topologically protected edge states can lead to important applications such as high‐transmittance waveguides,^2^, ^10^, ^14^, ^16^ robust photonic delay lines,^3^, ^13^ topological wave partition,^18^ robust photonic transport on nonplanar surfaces,^19^ topological lasers,^21^, ^22^ and topological quantum interfaces.^23^

The quest for topological 0D cavity modes in 2D electromagnetic‐wave systems,^24^, ^25^, ^26^ which could serve as an important ingredient to build‐up of robust electromagnetic‐wave/photonic devices, was unsuccessful until very recently.^28^, ^35^ Such an achievement was realized using the higher‐order topological insulators.^27^, ^28^, ^29^, ^30^, ^31^, ^32^, ^33^, ^34^, ^35^, ^36^, ^37^ Unlike the conventional *D*‐dimensional topological insulators which have (*D*−1)‐dimensional topological gapless boundary states, a *D*‐dimensional higher‐order topological insulator gives rise to (*D* − 2)‐dimensional (or even lower‐dimensional) topological gappless boundary states, in addition to the (*D* − 1)‐dimensional gapped boundary states, offering a paradigm beyond the conventional bulk‐boundary correspondence. Through the concept of higher‐order topological insulators, it has been demonstrated that topological 0D corner states can emerge in mechanical metamaterials,^27^ microwave circuits with lumped components,^28^ and coupled optical waveguides.^35^ However, in the electromagnetic‐wave/photonic systems, the microwave circuits with lumped components only work at low frequencies (normally up to several GHz), and the coupled optical waveguides have lattice constants much larger than the wavelength (*a* ≈ 40λ) and a small bandgap (2%). These realizations are hence unfavorable for future compact photonic devices.

Following the theory in a recent work,^38^ we here design and demonstrate a second‐order PTI which realizes photonic spin Hall effect through topological crystalline symmetry and gives rise to gapped edge states and topological corner states in surface‐wave photonic crystals (PhCs) with *a* ≈ 0.5λ. The surface‐wave PhC design provides a very large photonic bandgap (a bandwidth of 28% at the M point below the light‐line) as induced by multiple Bragg scatterings. The symmetry‐guided approach enables us to tune the photonic bandgap and band topology in both the bulk and the edges, simultaneously. In particular, the orthogonal edges in a finite square structure are described by 1D massive Dirac equations with tunable Dirac masses, which leads to topological localization of light at the corners connecting the orthogonal edges through the Jackiw–Rebbi mechanism^38^, ^39^ (see **Figure** **1**a). The gapped edge states as well as the topological corner states within the edge bandgap have been directly observed and characterized in our experiments. In comparison to the coupled optical waveguide arrays which have a large lattice constant (*a* ≈ 40λ) and small photonic bandgap (2%) and the microwave circuits with lumped components working at low frequencies, the present surface‐wave PhCs with a small lattice constant possess pronounced advantages^24^, ^25^, ^26^ such as large photonic bandgaps and miniature structures. The realization of the 0D topological corner states in PhCs provides an efficient way for scalable integration of cavity modes with identical and robust frequency for compact electromagnetic‐wave/photonic devices.

**Figure 1:**
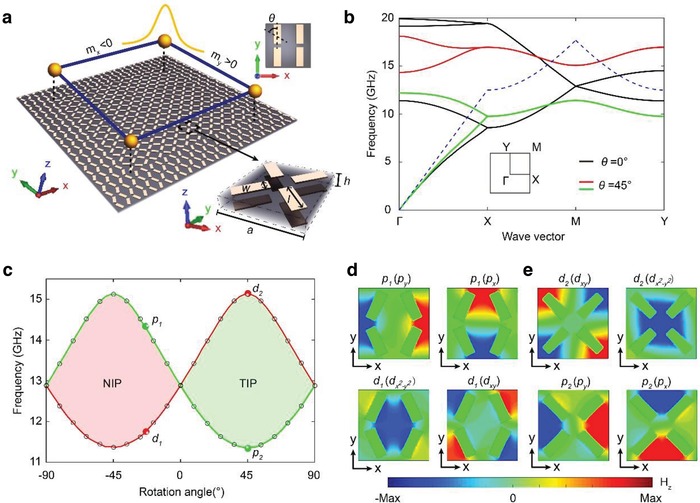
Second‐order PTI based on surface‐wave PhCs and its topological transitions. a) Schematic of topological corner states localized at the corner of the second‐order PTI. Based on the Jackiw–Rebbi mechanism, the opposite signs of the Dirac masses along the *x*‐ and *y*‐interfaces lead to the topological corner states. The upper inset shows the top view of the unit cell with rotation angle *θ* = 0°. The lower inset indicates the unit cell of the designed second‐order PTI with *θ* = 45°. The structure parameters are *h* = 2 mm, *w* = 1.92 mm, *l* = 5.04 mm, and *a* = 12 mm, respectively. The dielectric substrate has a relative permittivity of 3. b) Photonic band structures for *θ* = 0° (black curves) and *θ* = 45° (red and green curves), respectively. The blue dashed curve represents the light‐line in the air. The inset represents the Brillouin zone. c) Topological phases and the evolution of the photonic band edges at the M point with the rotation angle θ. The green and red curves represent the doubly degenerate *p* (dipole) and *d* (quadrupole) modes at the M point, respectively. d,e) Magnetic field profiles of the four eigenstates at the M point when *θ* = −25° (marked as *p*_1_ and *d*_1_) and 45° (marked as *d*_2_ and *p*_2_), respectively.

As depicted in Figure 1a, the design of the surface‐wave PhC consists of metallic patterns on both sides of a dielectric substrate in a square lattice with a lattice constant *a* = 12 mm. Each unit cell is composed of four metallic rectangle patterns on each side, with a width *w* = 1.92 mm and a length *l* = 5.04 mm. The photonic bands and band topology are determined by the rotation angle θ of the metallic rectangles (see the upper‐right inset of Figure 1a). Note that here we choose a unit cell which doubles the primitive unit cell. However, the chosen unit cell is the smallest unit cell that is compatible with the supercell structure for the edge and corner states^38^ studied in this work. In experiments, the designed surface‐wave PhCs are fabricated by printing double‐sided 0.035 mm thick copper cladding onto 2 mm thick F4B printed circuit boards (relative permittivity 3). Note that once the samples fabricated, the rotation angles of the metallic rectangles are fixed.

The surface‐wave PhC has a large topological bandgap. For *θ =* 45°, the frequency gap below the light‐line ranges from 11.5 to 15.2 GHz, leading to a bandwidth of 28% with a mid‐gap frequency of 13.3 GHz. The latter corresponds to a lattice‐constant/wavelength ratio of *a*/λ ≈ 0.5, indicating the subwavelength nature of the surface‐wave PhC. The 3D electromagnetic field profiles are presented in details in the Supporting Information.

The structure of the surface‐wave PhC has glide‐reflection symmetries in both the *x* direction *g_x_* = {*m_x_*|*τ_y_*} and the *y* direction *g_y_* = {*m_y_*|*τ_x_*}, where *m_x_ψ*(*x*, *y*, *z*) = ψ(−*x*, *y*, *z*), *τ_y_ψ*(*x*, *y*, *z*) = ψ(*x*, *y* + *a/*2, *z*), *m_y_ψ*(*x*, *y*, *z*) = ψ(*x*, −*y*, *z*) and *τ_x_ψ*(*x*, *y*, *z*) = ψ(*x* + *a/*2, *y*, *z*). Combining the glide‐reflection and time‐reversal symmetries, Kramer‐like double degeneracies exist at the boundaries of the Brillouin zone when *k_x_* = *π/a* or *k_y_* = *π/a* (the MX and MY lines),^38^, ^40^, ^41^ as shown in Figure 1b. When *θ =* 0° or 90°, a pair of doubly degenerate bands cross each other and form a fourfold Dirac degeneracy at the M point in the Brillouin zone at a frequency of 12.97 GHz (see the black curves in Figure 1b), which can also be explained by the Brillouin zone folding mechanism (see the Supporting Information). When θ deviates from 0° or 90°, the fourfold Dirac degeneracy splits into two pairs of doubly degenerate bands, and a photonic bandgap appears. For instance, the photonic band structure for *θ =* 45° is shown in Figure 1b (the red and green curves for the “conduction” and “valence” bands, respectively).

The evolution of the band edges at the M point with the rotation angle θ is shown in Figure 1c. These band edges consist of two pairs of photonic states of opposite parities (see Figure 1d): the even‐parity, quadrupole‐like modes (*d_x_* ^2^ *_−y_* ^2^ and *d_xy_*) and the odd‐parity, dipole‐like modes (*p_x_* and *p_y_*). When θ goes through 0° or 90°, the photonic bandgap experiences a parity switch, indicating a topological phase transition.^12^, ^38^ There are two topologically distinct phases: the normal insulator phase (NIP) and the topological insulator phase (TIP) (see the Supporting Information for the topological indices). The latter is a topological crystalline insulator which mimics the quantum spin Hall effect in photonic systems, as shown in the following.

Next, we investigate the edge states emerging at the interface between the NIP and TIP PhCs. Since the box‐shaped structure (Figure 1a) generally does not have *C*_4_ symmetry, the edge states at the *x*‐oriented and *y*‐oriented interfaces (shortened as the *x‐* and *y‐*interfaces) are generally different and should be investigated separately. In the following, we will study the photonic edge states at the *x‐* and *y*‐interfaces for two situations: First, for the box‐shaped structure with θ_1_ = −25° (NIP) and θ_2_ = 25° (TIP) (see **Figure** **2**a). Second, for the box‐shaped structure with θ_1_ = −25° (NIP) and θ_2_ = 50° (TIP) (see Figure 2b). Figure 2a demonstrates a case where the *x‐* and *y*‐interfaces preserve the glide symmetries (this holds whenever θ_2_ = −θ_1_), while Figure 2b demonstrates a case where such symmetries are broken on those edges.

**Figure 2:**
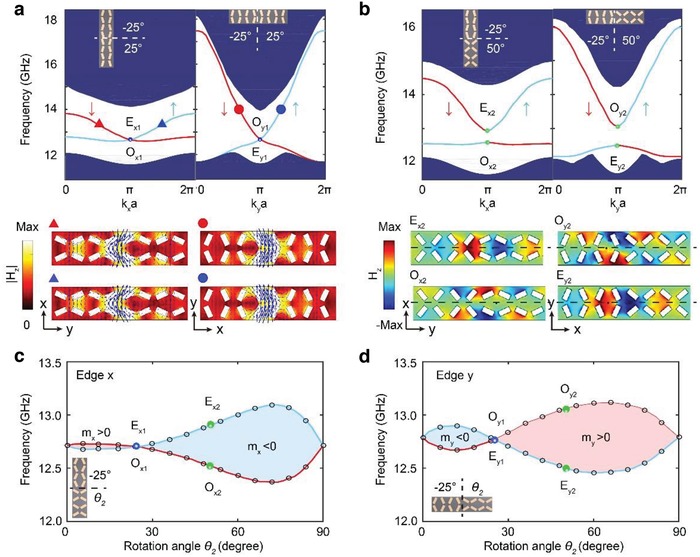
Edge states and their topological transitions in the surface‐wave PhCs. a) Edge states for the *x*‐ and *y*‐interfaces between the NIP (θ_1_ = −25°) and TIP (θ_2_ = 25°) PhCs, respectively. Here, the curves and blue regions denote the dispersions of edge and bulk photonic states, respectively. The blue (red) curves represent the pseudospin‐up↑ (pseudospin‐down↓) edge states. The upper insets indicate the schematics of the supercell. The lower panels represent the field profiles of the edge states labeled by the colored triangles/circles in the dispersions. The Poynting vectors (the blue arrows) indicate the finite orbital angular momenta for the edge states. b) Edge states at the *x*‐ and *y*‐interfaces between the NIP (θ_1_ = −25°) and TIP (θ_2_ = 50°) PhCs, respectively. Here, the lower panels represent the magnetic field distributions of the edge states at *k* = *π/a*. The black dashed lines indicate the symmetric axes. c,d) Topological transitions and Dirac masses of the edge states along *x*‐ and *y*‐interfaces as functions of the rotation angle θ_2_, with θ_1_ fixed to −25°. The blue (red) curves correspond to the even‐ (odd‐) edge modes at *k = π/a*. The blue (green) dots represent the cases of θ_2_ = 25° (50°). The insets show the schematics of the interface structures.

Figure 2a illustrates that the photonic edge states are time‐reversal symmetric helical edge states exhibiting pseudospin‐wavevector locking, where the pseudospins are emulated by the photonic orbital angular momentum as indicated by the winding of the Poynting vectors in the electromagnetic field profiles (see the Supporting Information for more details). These pseudospins confirm that we have realized a photonic analog of the quantum spin Hall effect. However, due to their bosonic nature, the photonic edge states are not protected by the time‐reversal symmetry. Consequently, the edge states can be gapped whenever the glide symmetries are absent at the edges (as exampled in Figure 2b), while the photonic edge states are described by 1D massive Dirac equations. These gapped edge states can be described by the Hamiltonians $H_{\alpha }=v_{\alpha }\left(k_{\alpha }-\frac{\pi }{a}\right)\sigma_{z}+m_{\alpha }\sigma_{y}$ with α = *x*, *y* for the *x‐* and *y*‐interfaces, where *v*_α_ is the group velocity of the edge states; σ_*z*_ = 1 and −1 represent the pseudospin‐up and ‐down modes.^38^ The Dirac masses *m*_α_ are determined by half of the frequency difference between the even and odd modes at $k_{\alpha }=\frac{\pi }{a}$ (α = *x*, *y*). The topology of the 1D photonic edge states is characterized by the sign of the Dirac masses. Parity switch in the edge states thus signals the topological transition in the edge states.

The Dirac masses of the edge states at *x*‐ and *y*‐interfaces, *m_x_* and *m_y_*, are generally different and can be controlled by the rotation angles θ_1_ and θ_2_. In Figure 2c,d, we show the Dirac mass as a function of θ_2_ for 0° ≤ θ_2_ ≤ 90°, with θ_1_ fixed at −25°. There are three topological transition points for the edge states, θ_2_ = 0°, 25°, and 90°, in those phase diagrams. The transition points θ_2_ = 0° and 90° are associated with the topological transitions of the bulk photonic bands. In contrast, the transition point θ_2_ = 25°, with both *m_x_* and *m_y_* equal to zero, is solely due to the edge (as shown in Figure 2c), resulting from the restoration of the glide symmetries at the two interfaces. Interestingly, despite the topological transition at θ_2_ = 25°, the signs of *m_x_* and *m_y_* remain opposite before and after the transition, which manifests the stability of the higher‐order band topology.

In the supercell structure illustrated in Figure 1a, the opposite signs of *m_x_* and *m_y_* lead to the formation of 0D photonic states localized at the four corners connecting the *x*‐ and *y*‐interfaces, due to the Jackiw–Rebbi mechanism.^38^, ^39^ Moreover, each *x*‐interface or *y*‐interface can be regarded as a 1D system with mirror symmetry, and, therefore, the parities of edge states at the center and boundary of the 1D edge Brillouin zone determine the corresponding Zak phases.^38^, ^42^ The trivial Zak phase at one interface and nontrivial Zak phase at another interface guarantee the existence of topological corner states.^30^ Hence, in our system the 2D bulk topology results in the 1D edge states, while the topology of the gapped 1D edge states leads to the 0D corner states. This manifestation of bulk‐edge correspondence in a hierarchy of dimensions reveals a hallmark feature of the second‐order topology.^38^

In the experiments, we fabricate two different square‐shaped super‐structures with both NIP and TIP PhCs. The first sample consists of the PhC with θ_2_ = 25° (TIP) surrounded by the PhC with θ_1_ = −25° (NIP) (see **Figure** **3**a). The edge states are excited by a dipole source near the bottom of the sample located at the position labeled by the red star in Figure 3a. The response at the *x*‐edge (*y*‐edge) is detected by a probe located at the blue (green) dot. The response of the bulk is measured by another probe at the center of the TIP PhC (see details in the Supporting Information). The measured (normalized) |*H*_z_|^2^ field intensities at those detection positions are shown in Figure 3b. One can see that the transmission of the bulk (the gray region) are very low in the frequency range from 12.5 to 14 GHz, in consistency with the bulk bandgap. Within this frequency range, the responses for the edges along both the *x*‐ (the blue region) and *y*‐ (the green region) interfaces are much stronger than the bulk, indicating the edge states within the bulk bandgap. Both the *x*‐ and *y*‐edges have continuous (gapless) responses, indicating their gapless spectrum, in agreement with the edge dispersion in Figure 2a. The electromagnetic field profile measured directly by a near‐field scanning system (see the Supporting Information) is shown in Figure 3c. The measured field profile and the corresponding simulation (Figure 3d) indicate light flow along the edges since the exciting frequency 13.24 GHz is in the bulk bandgap. In addition, more scanned fields at different frequencies can be found in the Supporting Information. There are slight differences between the numerical and experimental results, which may be due to the limited fabrication and measurement accuracy, such as the geometry deviation of the fabricated sample from the ideal model and tremble of moving receive antenna in the measurement.

**Figure 3:**
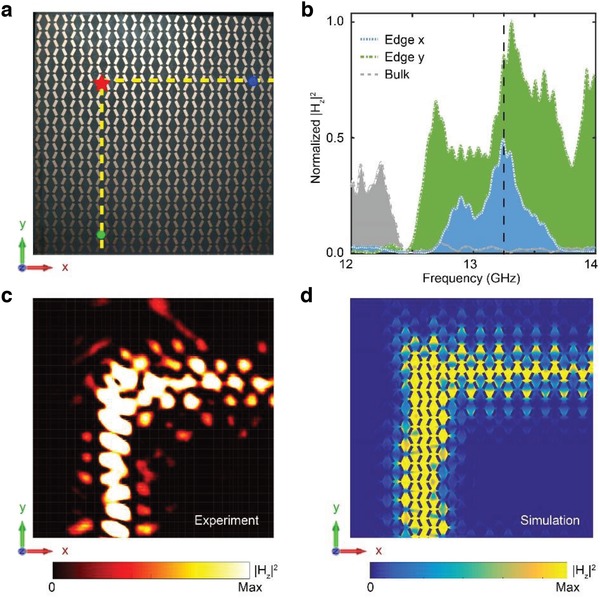
Experimental observation of gapless topological edge states in the surface‐wave PhCs. a) Perspective‐view photograph of the sample (only the upper‐left quarter of the structure is shown), composed of the PhC with θ_2_ = 25° (TIP, in the lower‐right side of the yellow dashed lines) and the PhC with θ_1_ = −25° (NIP, in the other region). The red star represents the location of the point source. The green and blue dots denote the locations of the probes. b) Normalized magnetic field intensity |*H*_z_|^2^ at the two edge probes (the blue and green regions for the *x*‐ and *y*‐edges, respectively) and at the bulk probe (located at the center of the sample). c,d) Measured and simulated magnetic field intensity distribution |*H*_z_|^2^ over the sample at 13.24 GHz (marked by the black dashed line in Figure 3b) excitation, respectively.

We further measure a sample consisting of a TIP PhC with θ_2_ = 50° surrounded by an NIP PhC with θ_1_ = −25°, as illustrated in **Figure** **4**a. We measure the responses of the edge and bulk states in similar means as in Figure 3. The results in Figure 4b reveal that both the *x*‐ and *y*‐edges have gapped photonic spectrum within the bulk bandgap (i.e., from 12.5 to 14 GHz). We also measure the response of the corner by placing a probe with two unit‐cell distance away from the dipole source. The corner response clearly indicates a strong and sharp peak at 12.71 GHz within the spectral gaps of both the *x*‐ and *y*‐edges (their common frequency gap ranges from 12.6 to 12.8 GHz). Such a sharp resonance indicates the emergence of the topological corner modes. The spectral fingerprints with edge states in the bulk bandgap and corner states in the edge bandgap is a hallmark feature of second‐order topological insulators.

**Figure 4:**
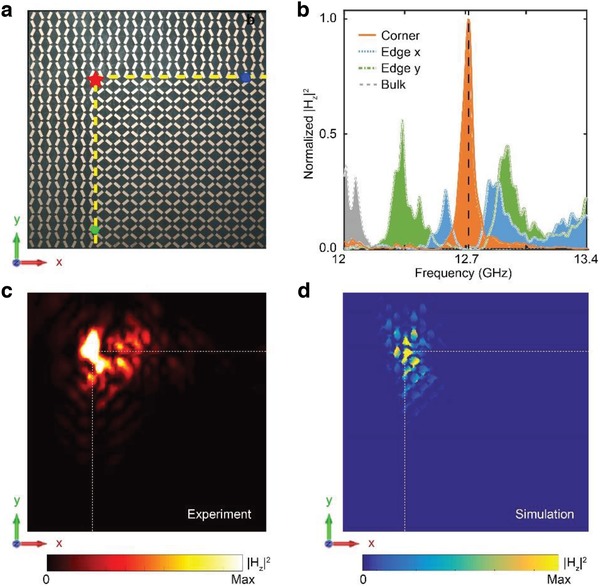
Experimental observation of topological corner states in surface‐wave PhCs. a) Perspective‐view photograph of the experimental sample (only the upper‐left quarter of the structure is shown), composed of a PhC with θ_2_ = 50° (TIP) (at the lower‐right side of the yellow dashed lines), surrounded by the PhC with θ_1_ = −25° (NIP). The red star represents the location of the point source. The green and blue dots denote the locations of the probes. b) Normalized magnetic field intensity |*H*_z_|^2^ at the two edge probes (the blue and green regions for the *x*‐ and *y*‐edges, respectively) and at the bulk probe (located at the center of the sample). c,d) Measured and simulated magnetic field intensity distribution |*H*_z_|^2^ over the sample at 12.71 GHz (marked by the black dashed line in Figure 3b) excitation, respectively. The interface between the TIP and the NIP PhCs is labeled by the white dashed lines.

The corner resonance is further studied by measuring its electromagnetic profile using the near‐field scanning method. The measurement results, shown in Figure 4c, is consistent with the simulated field profile in Figure 4d. More scanned field distributions and the corresponding simulated ones for counterparts at other frequencies can be found in the Supporting Information. The robustness of the topological corner states is examined numerically by simulating the frequency‐domain response of the corner states and studying the frequency stability against deformations and defects (see the Supporting Information). Our numerical simulations verify that the frequency of the topological corner states is robust against certain types of disorders and deformations.

Exploiting surface‐wave PhCs, we realized experimentally a higher‐order photonic topological insulator with edge states and topological corner states. The concurrent emergence of gapped edge states within the bulk bandgap and topological corner states within the edge bandgap demonstrates the higher‐order topology in our PhCs. In particular, the strongly confined topological corner states provide an efficient approach toward scalable integration of degenerate cavity modes in photonic devices. Besides, our surface‐wave PhCs offer a versatile platform for the realization of higher‐order topology through multiple Bragg scatterings which can yield photonic bandgaps much larger than those in the coupled optical waveguide arrays or other analogs with perturbative tight‐binding couplings. Although our experiments are conducted at microwave frequencies, our design can work up to terahertz frequencies by scaling down the current structure. The symmetry‐guided design principle can also be applied to dielectric materials, which are available from microwave up to optical regime (see the Supporting Information). Our study thus opens a pathway toward higher‐order photonic topology with large bandwidths for future electromagnetic‐wave/photonic applications.

After the submission of this work, we become aware of several independent works, which report on realization of higher‐order photonic topological states based on photonic crystals in a generalized Su‐Schrieffer‐Heeger model,^43^, ^44^, ^45^ or based on optical waveguide arrays in a breathing kagome lattice model,^46^ or realization of photonic topological quadrupole phases.^47^

## Conflict of Interest

The authors declare no conflict of interest.

## Supporting information

Supporting Information
