# Supplementary material for: Higher‐Order Topological States in Surface‐Wave Photonic Crystals
Source: Adv Sci (Weinh). 2020 Jan 27;7(6):1902724. doi: 10.1002/advs.201902724 (PMC7080542; doi:10.1002/advs.201902724)
Supplement: Supplementary file 1 — Supporting Information [file ADVS-7-1902724-s001.pdf]

Copyright WILEY-VCH Verlag GmbH & Co. KGaA, 69469 Weinheim, Germany, 2020.

ADVANCED SCIENCE
Open Access

Supporting Information

for *Adv. Sci.,* DOI: 10.1002/advs.201902724

Higher-Order Topological States in Surface-Wave Photonic Crystals

Li Zhang, Yihao Yang, Zhi-Kang Lin, Pengfei Qin, Qiaolu Chen, Fei Gao, Erping Li, Jian-Hua Jiang,* Baile Zhang, and Hongsheng Chen**

Supplementary Information

Higher-order topological states in surface-wave photonic crystals

Li Zhang[1,2,#], Yihao Yang[3,4,#, *], Zhi-Kang Lin[5], Pengfei Qin[1,2], Qiaolu Chen[1,2], Fei Gao[1,2], Erping Li[1,2], Jian-Hua Jiang[5,*], Baile Zhang[3,4,], and Hongsheng Chen[1,2,*]

[1]Interdisciplinary Center for Quantum Information, State Key Laboratory of Modern Optical Instrumentation, College of Information Science and Electronic Engineering, Zhejiang University, Hangzhou 310027, China.

[2]Key Laboratory of Advanced Micro/Nano Electronic Devices & Smart Systems of Zhejiang, The Electromagnetics Academy at Zhejiang University, Zhejiang University, Hangzhou 310027, China.

[3]Division of Physics and Applied Physics, School of Physical and Mathematical Sciences, Nanyang Technological University, 21 Nanyang Link, Singapore 637371, Singapore.

[4]Centre for Disruptive Photonic Technologies, The Photonics Institute, Nanyang Technological University, 50 Nanyang Avenue, Singapore 639798, Singapore.

[5]School of Physical Science and Technology, and Collaborative Innovation Center of Suzhou Nano Science and Technology, Soochow University, 1 Shizi Street, Suzhou 215006, China.

# These authors contributed equally to this work.

[*] *yang.yihao@ntu.edu.sg* (Yihao Yang); *jianhuajiang@suda.edu.cn* (Jian-Hua Jiang); *hansomchen@zju.edu.cn* (Hongsheng Chen).

Supplementary Note 1: Surface wave on the surface-wave photonic crystals

Here, Figure S1 exhibits the perspective and side views of magnetic field distributions of the eigenstates at the M point when $\theta = 0°$. One can see that the energy is strongly confined around the surface and dramatically decays into the air. Note that the bands we study are under the light lines, revealing the surface-wave nature of modes.

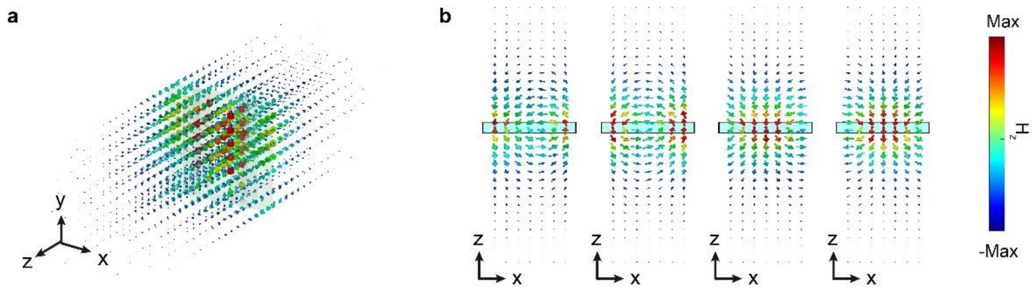

Figure. S1. Field distributions of eigenstates. a, b, Perspective view of one selected magnetic field pattern and side view of magnetic field patterns of the four eigenstates at the M point when θ = 0°, respectively.

Supplementary Note 2: Zone folding mechanism

As depicted in Figure S2a, the unit cell of the surface-wave photonic crystal (PhC) is a larger square non-primitive unit cell (marked as II), composed of four smaller square primitive unit cells (marked as I). Since the non-primitive unit cell is four times larger than the primitive one (Figure S2a), the first Brillouin zone (FBZ) of the non-primitive unit cell is a quarter of the FBZ of the primitive unit cell (Figure S2b). By comparing unit cells and their associated FBZs, we can easily obtain the dispersions of the non-primitive unit cell based on zone folding mechanism[1, 2] (Figure S2c), which indicate that the non-primitive FBZ ($\Gamma_{II}X_{II}M_{II}Y_{II}$) can be formed by folding the primitive FBZs ($X_{II}X_{I}AM_{II}$, $M_{II}AM_{I}B$, $Y_{II}M_{II}BY_{I}$, and $\Gamma_{I}X_{II}M_{II}Y_{II}$).

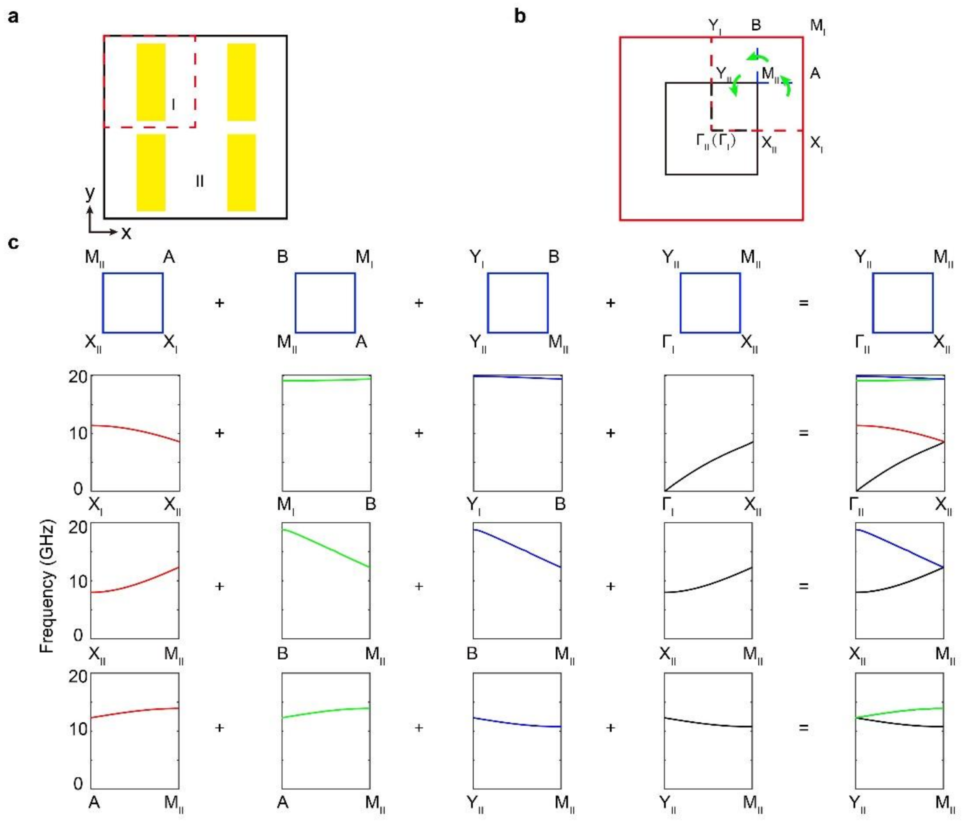

Figure. S2. Zone folding mechanism. a, Schematic of the non-primitive unit cell (II) and primitive unit cell (I). **b**, Corresponding FBZs of the non-primitive and primitive unit cell. **c,** Band folding processes.

Supplementary Note 3: Topological indices

In this section, we study the topological indices for the PhC in the framework of topological crystalline insulators[6]. Generically, we can distinguish nontrivial topological classes arising from the C_n symmetry by comparing the representations of symmetry at high symmetry points (HSPs) of the occupied bands in the FBZ[3-5]. In this work, the unit cell belongs to C_2 system for each rotation angle, so that the eigenstates of HSPs (Γ, X, M and Y) are studied. The eigenvalues of rotation operator at the HSP Π can be defined as $\Pi_p^{(2)} = e^{2\pi i(p-1)/2}$, where, p = 1, 2 represent two different rotation representations, and Π refers to HSPs Γ, X, M and Y. The topological classes of crystals with rotation symmetry C_2 are given by the indice $\chi^{(2)}$, as follow[3],

$$\chi^{(2)} = ([X_1^{(2)}],[Y_1^{(2)}],[M_1^{(2)}]) \quad (1)$$

Where $[\Pi_1^{(2)}] = \#\Pi_1^{(2)} - \Gamma_1^{(2)}$ (Π = Γ, X, M and Y), $\#\Pi_1^{(2)}$ and $\#\Gamma_1^{(2)}$ are the number of energy bands below the band gap with eigenvalue $\Pi_1^{(2)}$ and $\Gamma_1^{(2)}$, respectively. As shown in Figure S3, each rotation eigenvalue is provided at the corresponding HSPs,

which can be easily attached by observing the field patterns or the phase distributions of the wavefunctions. According to Eq. 1, we can calculate the topological indices, that is, $\chi^{(2)}$ = (-1, -1, 0) for unit cells with negative rotation angles, and $\chi^{(2)}$ = (-1, -1, -2) for unit cells with positive rotation angles. Due to the different indices $\chi^{(2)}$, the C_2 symmetric configurations with different signs of θ belong to different topological classes[3-5]. Here, we define the two topologically distinct phases as the normal insulator phase (NIP) and the topological insulator phase (TIP), respectively.

In addition, the value of Wannier polarization[3], which is defined modulo 1, is written in terms of the invariants in Eq. (1) as

$$\begin{aligned} p_1 &= \frac{1}{2}([Y_1^{(2)}]+[M_1^{(2)}]) \\ p_2 &= \frac{1}{2}([X_1^{(2)}]+[M_1^{(2)}]) \end{aligned} \quad (2)$$

The Wannier polarization for both photonic crystals is **P** = (1/2, 1/2). When two photonic crystals with the same Wannier polarization are placed together, such a Wannier polarization will not exhibit any physical consequence. This situation is, in fact, equivalent to the situation when two photonic crystals with vanishing Wannier polarization are placed together.

Finally, the secondary topological index, the nominal corner charge which is also defined modulo 1, is given by the above topological invariants[3], as follow,

$$Q^{corner} = \frac{1}{4}(-[X_1^{(2)}]-[Y_1^{(2)}]+[M_1^{(2)}]) .$$

Using the above equation, one finds that Q^{corner} = 1/2 for NIP and Q^{corner} = 0 for TIP. The distinct corner charge implies the emergence of the corner states when the NIP and TIP photonic crystals are placed together to form a large structure with four corners, which is consistent with our findings in both simulations and experiments.

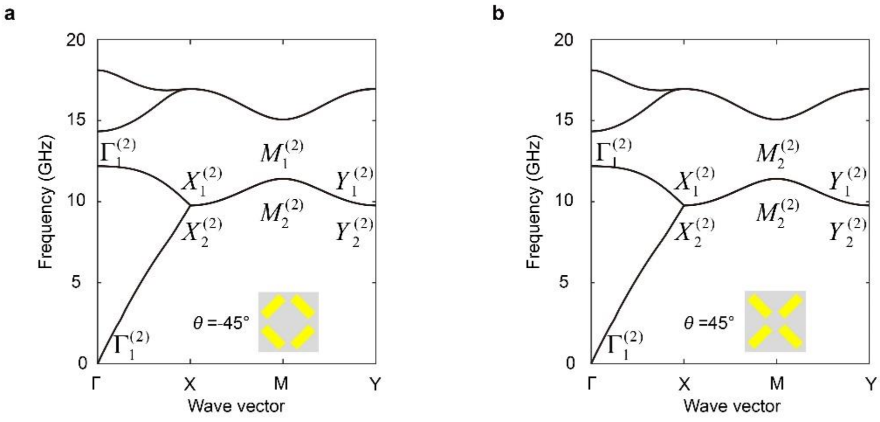

Figure. S3. The band structures for NIP and TIP. a, The band structure for NIP (θ = -45°). **b**, The band structure for TIP (θ = 45°). Here, eigenvalues at the high symmetric points determine the topological invariants.

Supplementary Note 4: Pseudospin-up and pseudospin-down edge states

As shown in Figure. S4, we check the real-space distributions of the Poynting vectors around horizontal and vertical interfaces, which describe upward and downward energy flows for the pseudospin-up and pseudospin-down states, respectively. It can also be observed that the field distributions are highly concentrated on horizontal and vertical interfaces. Note that this phenomenon is similar to quantum spin Hall effect in photonic systems[6, 7].

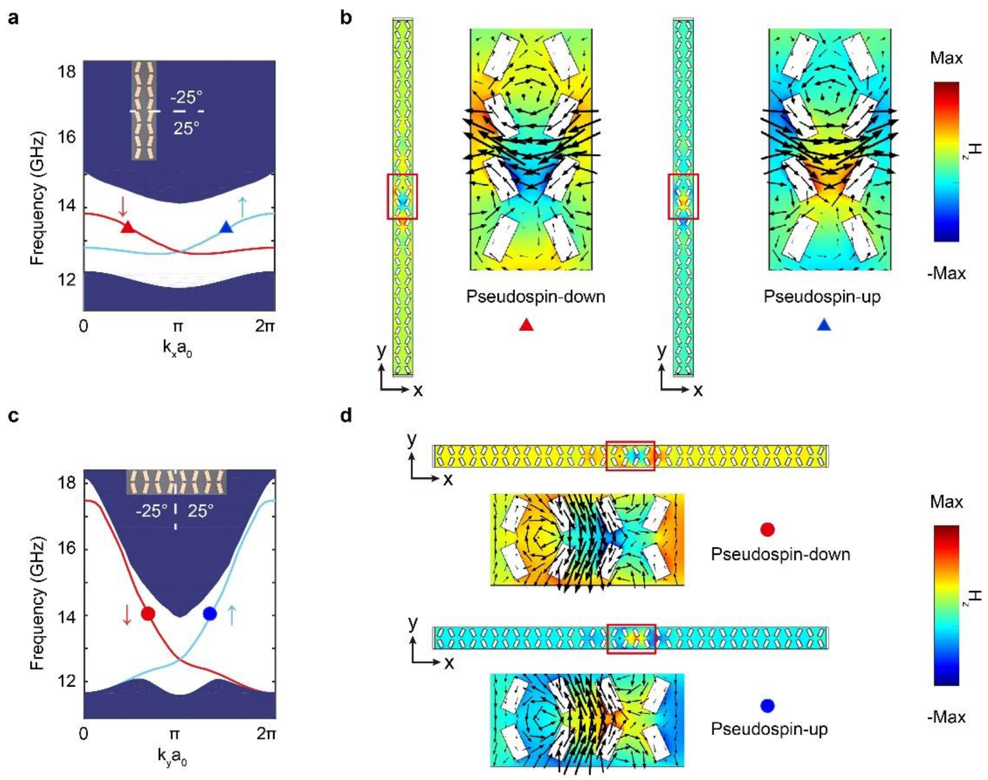

Figure. S4. Dispersion relations and field distributions of horizontal and vertical interfaces. a, c, Edge states at horizontal and vertical interfaces that consist of NIP (θ_1 = -25°) and TIP (θ_2 = 25°) PhCs, respectively. **b, d,** Red and blue triangles/circles label the field distributions of pseudospin-down and pseudospin-up states, respectively. Black arrows represent the real-space distributions of the Poynting vectors.

Supplementary Note 5: More experimental results on the measurement of gapless topological edge states

In this section, we show more experimental results for the sample in Figure 3a. Here, Figure S5 illustrates three magnetic field intensity distributions at the frequencies of 12.6 GHz, 13.24 GHz, and 13.85 GHz, respectively. As shown in Figure S5a, only edge state exists along the *y*- interface at 12.6 GHz. When increasing the frequency of excitation to 13.24 GHz, the edge states will transport along both *x*- and *y*- interfaces. However, the field distribution returns to edge state along the *y*- interface at 13.85

GHz. Obviously, the experimental observations (Figure S5a-c) are in good agreement with the numerical results (Figure S5d-f). Moreover, these field distributions are almost identical to the measured transmission spectra (shown in Figure 3b). And these results can also be theoretically explained according to the edge states dispersions as illustrated in Figure 2a.

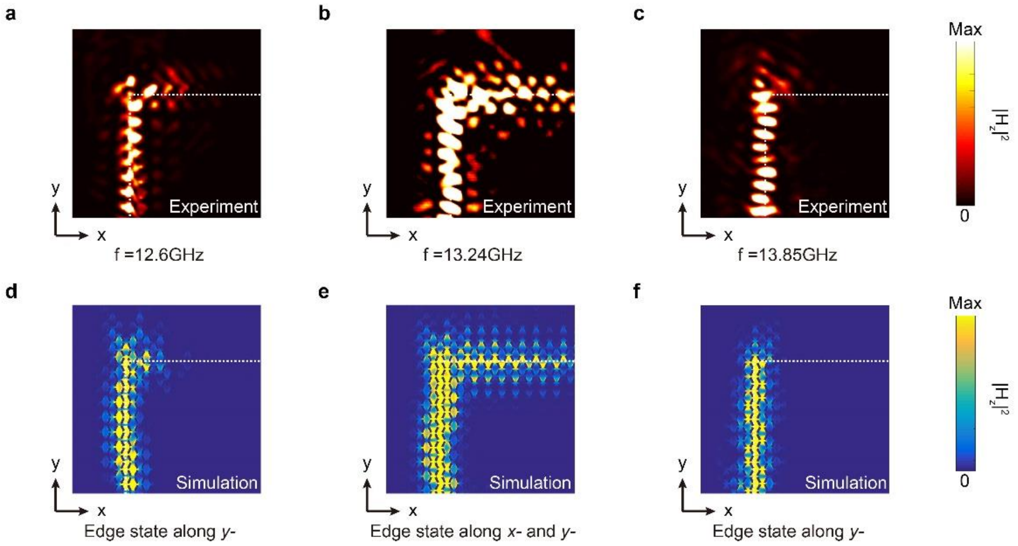

Figure. S5. More experimental results for the sample in Figure 3(a). a-f, Measured and simulated magnetic field intensity distribution $|H_z|^2$ over the sample at 12.6 GHz, 13.24 GHz and 13.85 GHz, respectively.

Supplementary Note 6: More experimental results on the measurement of topological corner state

In this section, we present more scanned experimental results for the sample shown in Figure 4. For instance, as illustrated in Figure S6a, there only exist edge state along the *y*- interface at 12.33 GHz. In Figure S6b, the edge state along the *x*- interface is presented at 12.59 GHz. Significantly, a corner state exists at 12.71 GHz. When the frequency increases to 12.94 GHz, edge states along both *x*- and *y*- interfaces will exist. And the field distribution returns to edge state along the *y*- interface at 13.85 GHz, as shown in Figure S6e. It is obvious that the experimental observations (Figure S6a-e) are in good agreement with the numerical results (Figure S6f-j). These field distributions are consistent with the measured transmission spectra (shown in Figure 4b). These results can be explained according to the edge states dispersions as illustrated in Figure 2b. The field distributions emerge as a 1D edge state, a 0D corner state and 1D edge state, when the frequency increases.

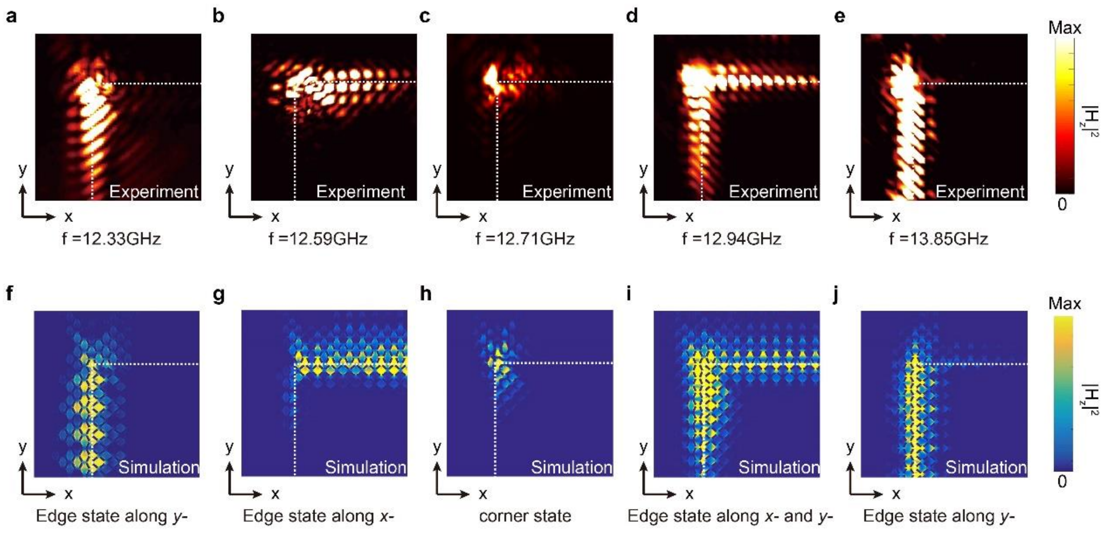

Figure S6. More experimental results for the sample in Figure 4(a). a-j, Measured and simulated magnetic field intensity distribution $|H_z|$ over the sample at 12.33 GHz, 12.59 GHz, 12.71 GHz, 12.94 GHz and 13.85 GHz, respectively.

Supplementary Note 7: Measured magnetic field distribution of the topological corner state

Here, we present the measured magnetic field (H_z) distribution of the topological corner state. As illustrated in Figure S7a, it is obvious that the experimental result is in good agreement with the simulated one. Note that the circular patterns are radiative noise.

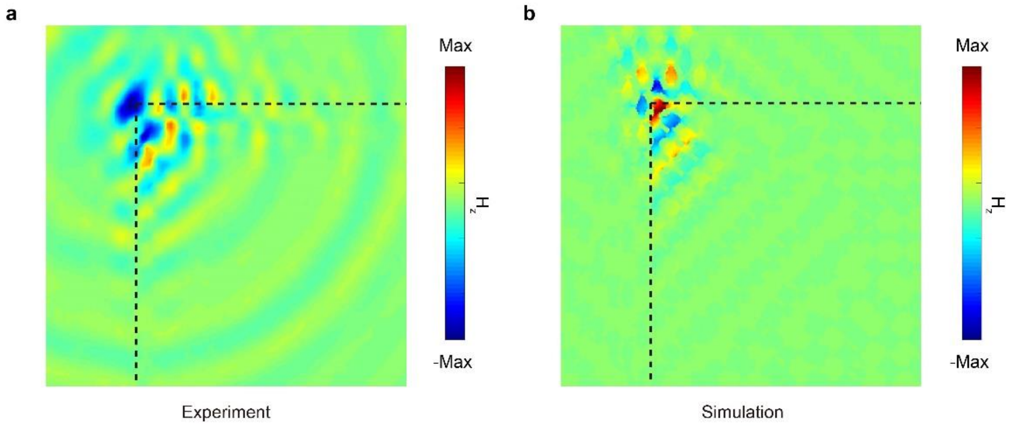

Figure S7. Measured magnetic field distribution of the topological corner state. a, b, Measured and simulated magnetic field distribution H_z over the sample at 12.71 GHz, respectively.

Supplementary Note 8: Eigenfrequencies and eigenmodes

In this section, we numerically calculate eigenfrequencies and eigenmodes for the

box-shaped structure in Figure 1a (with relative permittivity 3.5), with rotation angle θ_1 = -25° and θ_2 = 50°. As shown in Figure S8a, there are four modes (red dots) away from other modes. The field distributions of the four eigenmodes are exhibited in Figure S9b, which clearly indicate the energy is highly localized at the corners, revealing the existence of topological corner states.

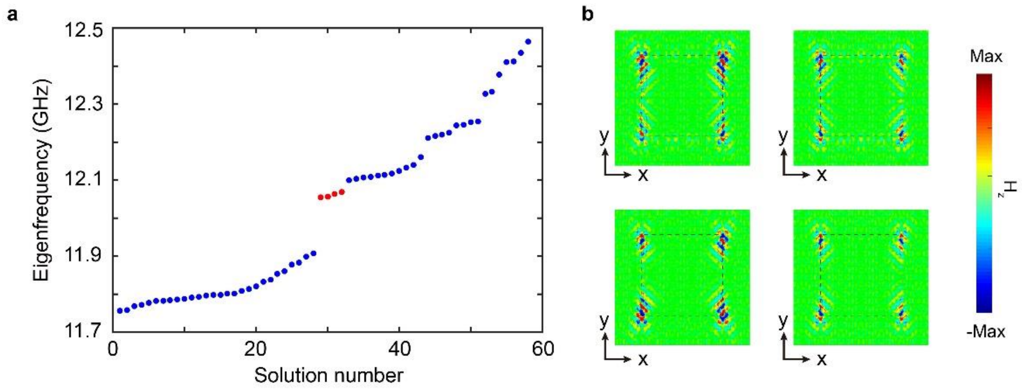

Figure S8. Numerically calculated eigenfrequencies and eigenmodes. a, Numerically calculated eigenfrequencies for the box-shaped structure in Figure 1a. **b**, Magnetic field distributions of the four eigenmodes (red dots in **a**). The dashed lines represent the interfaces between TIP and NIP PhCs.

Supplementary Note 9: Robustness of the mid-gap topological corner states

In this section, we numerically verify the robustness of topological corner states by introducing imperfections. There are two main methods to disturb the configuration: adjusting (i) rotation angle and (ii) size of the rectangle patterns. For the first approach, the unit cell at the corner (red pattern in Figure S9a) or unit cell near the corner (Figure S9b) is perturbed by rotating 5° clockwise. For the second method, we change the size of the unit cell at the corner (Figure S9c) or near the corner (Figure S9d), i.e., increasing the length of the metallic patterns from 5.04 mm to 5.24 mm. The field distributions (the middle figures) show the existence of robust topological corner states. Then we increase the disturbing strength of the several unit cells by rotating red patterns 5° clockwise and blue patterns 10° anticlockwise, as shown in Figure S9e. It is obvious that the corner states are robust even in the presence of large perturbation. Additionally, we plot the transmissions along the corners, which are shown on the right. Despite the frequency shifting, the peaks in the diagrams reveal the existence of robust corner states. Note that the excitation port is set at one corner, and the transmissions are obtained by placing probes at the corner, the horizontal and vertical interfaces.

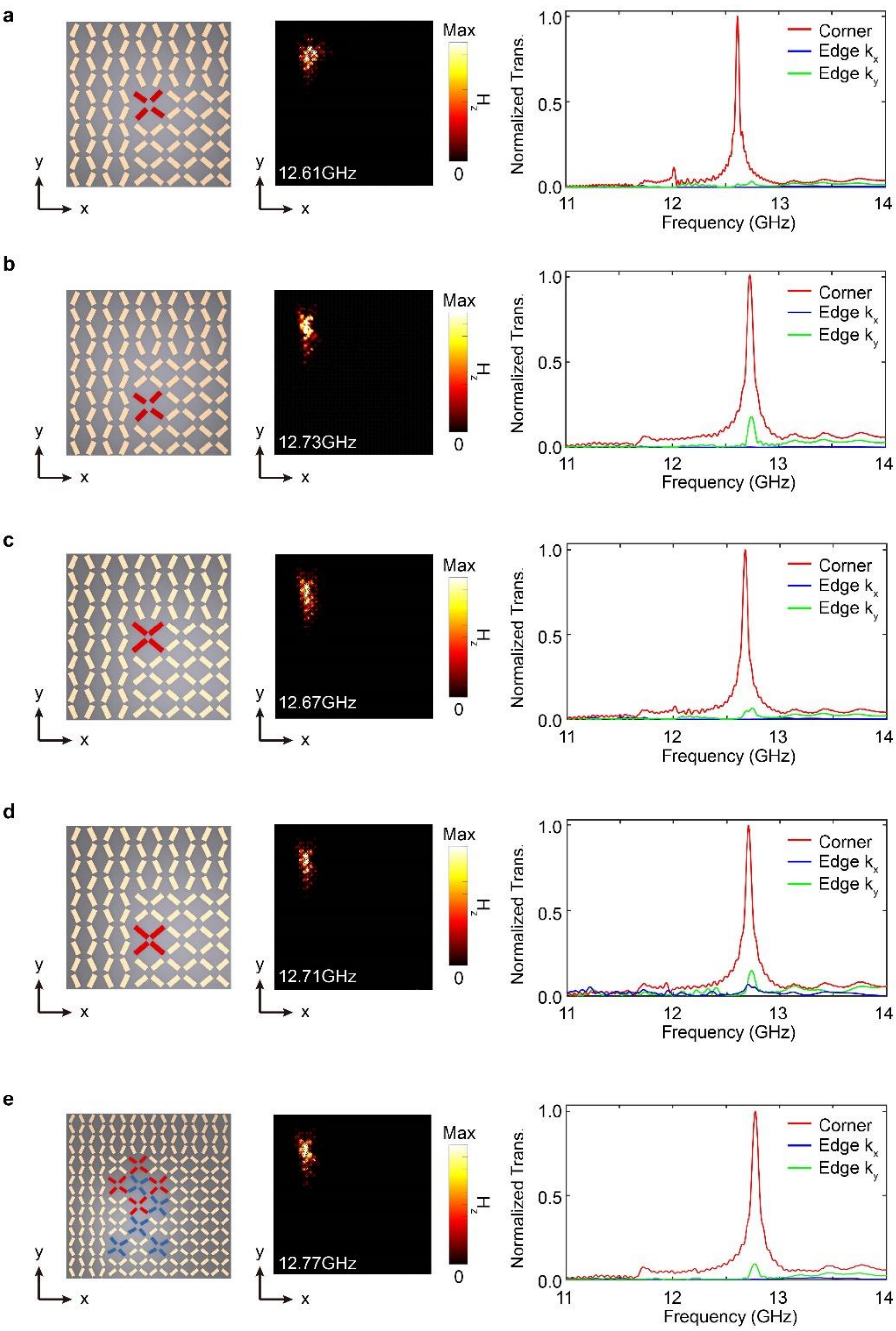

Figure S9. Field distributions and transmission after introducing perturbations. a-e, The figures on the left depict a part of the schematic consisting of 8×8 unit cells after perturbations, i.e. rotating the unit cell at the corner **a** or near the corner **b** for 5°, increasing the length of the metallic patterns of unit cells at the corner **c** or near the corner **d** from 5.04 mm to 5.24 mm, and rotating the unit cells near corner for 5° and -10° **e**. The red and blue squares highlight the perturbations. The middle figures represent the field distributions of corner states. The figures on the right indicate the transmissions of corner states and edge states for horizontal and vertical

interfaces, where the peaks indicate the corner states.

Supplementary Note 10: Higher-order topological states in all-dielectric photonic crystals

Here, we show a design of all-dielectric higher-order photonic topological insulators based on the design principle in this work.

As illustrated in Figure S10a, the newly-designed all-dielectric (with relative permittivity 25, such as PbTe at mid-infrared frequencies[8]) unit cell is proposed, which is composed of four elliptic rods and has glide-reflection symmetries in both *x*- and *y*- directions. Here, the grey region represents dielectric structure and the blue area indicates vacuum. The elliptic silicon rod has a major axis $b_1 = 0.3a$, a minor axis $b_2 = 0.05a$. Here, a is the lattice constant. When tuning the rotation angle of the elliptic rods, the band structure changes. For instance, for $\theta = 45°$, the band structure and the eigenmodes at the M point are shown in Figure S10b, which have the same characteristics as those in the main text. Then we study the photonic edge states along both *x*- and *y*- interface for the PhCs with $\theta_1 = -52°$ and $\theta_2 = 48°$, as shown in Figure S10c. Due to the absence of glide symmetries, the edge states are gapped, which ensure the existence of the 0D corner state. Next, we design a box-shaped sample consisting of a PhC with $\theta_2 = 48°$ surrounded by a PhC with $\theta_1 = -52°$, as illustrated in Figure S10a. We numerically calculate eigenfrequencies of the sample, as illustrated in Figure S10d. Obviously, there are four modes away from each other, and the eigenmodes are highly confined around the corners, verifying the existence of 0D corner states.

In conclusion, based on the design principle in this work, the higher-order topological phases can also be realized in all-dielectric PhCs which are available from microwave up to optical frequencies.

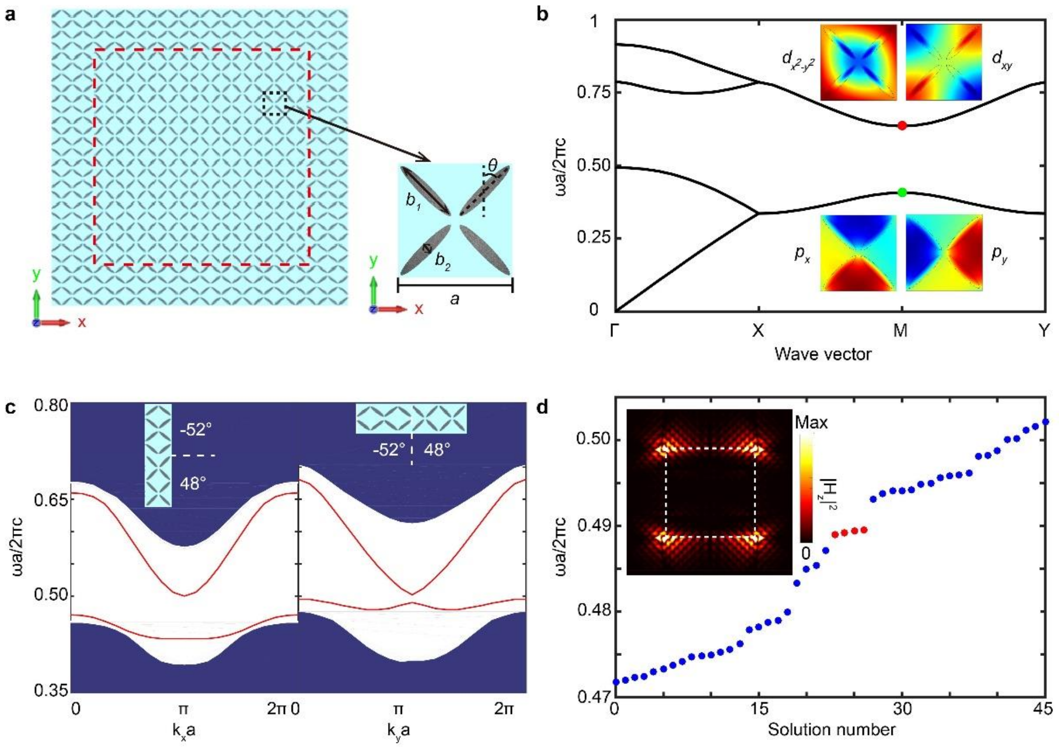

Figure S10. Design of an all-dielectric photonic-crystal-based higher-order photonic topological insulator. a, Schematic of the all-dielectric high-order photonic topological insulator. Inset represents a unit cell. The grey area is the dielectric material (with relative permittivity 25) and the blue region represents vacuum. The structure parameters are b_1 = 0.3*a*, b_2 = 0.05*a*, respectively. Here, *a* is the lattice constant. **b,** Photonic band structures for θ = 45°. The inset figures illustrate magnetic field distributions of the four eigenmodes at the M point. **c,** Edge states at the *x*- and *y*- interfaces between the PhCs with θ_1 = -48° and θ_2 = 52°, respectively. **d,** Numerically-calculated eigenfrequencies for the box-shaped structure in Figure S1**a**. The inset figure illustrates the magnetic field intensity distribution of the eigenmodes (red dots). The white dashed lines represent the interfaces between different kinds of PhCs.

Supplementary Note 11: Methods

Numerical simulations.

We perform simulations using the commercial software Computer Simulation Technology (CST) Microwave Studio. In the simulations, the metallic patterns are considered as perfect electric conductor (PEC). Periodic boundary is applied along the *x*- and *y*- directions in eigenmode solver to obtain the dispersion relations and magnetic field patterns. We use open boundary conditions in time domain solver to gain magnetic field distributions along the interfaces. Meanwhile, we use the commercial software COMSOL Multiphysics to get the Poynting vectors.

Experiments. The PCs are fabricated with printed circuit board (PCB) technology,

and double-sided 0.035-mm-thick copper (dimensional tolerance +/-0.1mm) is cladded onto 2-mm-thick F4B PCB (relative permittivity 3.00+/-0.05, thickness tolerance +/-0.01mm). The experimental setup is shown in Figure S11. To prevent waves from leaking into the air that might influence the results, we set the source at the bottom of the PCs, marked with a red star as shown in Figure 3a and Figure 4a. We place the detector on the top of the sample. For the transmission measurement of corner states, the probe is placed two-unit-cell distance away from the source. For the transmission measurement of edge states along the horizontal and vertical interfaces, the probe is set at five random points along the black dashed lines along *x*- and *y*-directions, for example, green and blue dots shown in Figure 3(a) and Figure 4(a). For transmission measurement of bulk states, we place the probe at five random points at the center of the sample. The measuring point may have a little shift from the precise point. The testing values in transmission spectra depicted in Figure 3(b) and Figure 4(b) are averaged and then normalized by the maximum values so that the difference between topological states can be clearly observed. The experimental results in Figure 3(c) and Figure 4(c) are obtained by scanning the magnetic field distributions of the whole PCs. The probing antenna that is placed above the top of the samples for 1mm, is controlled by a near-field scanning system. Here, we set the stepping as 2mm.

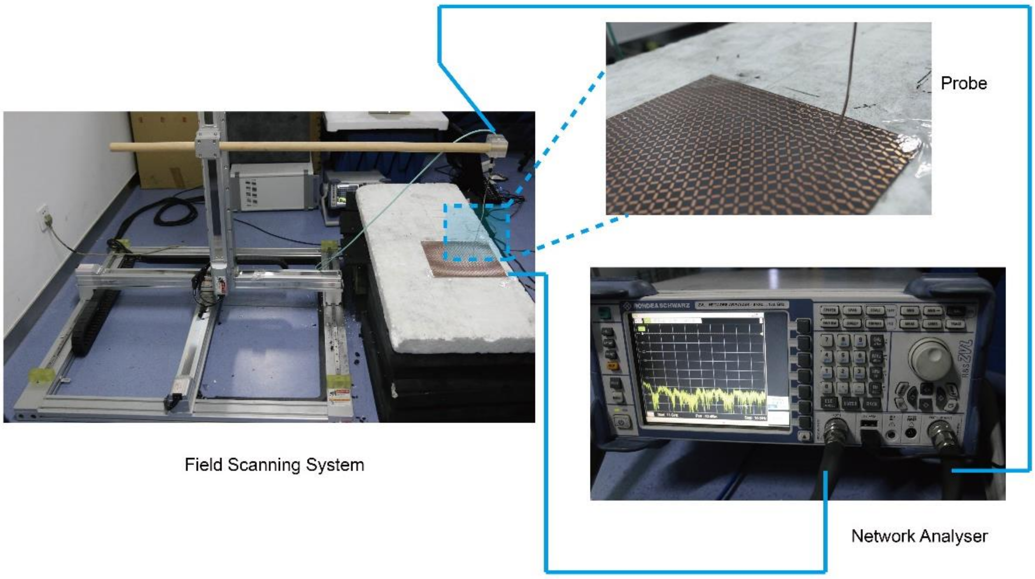

Figure S11. Experimental setup. Experimental setup includes a fabrication sample, a vector network analyzer, and a near-field scanning system. The vector network analyzer provides transmission of electromagnetic waves through the fabricated sample by placing the source on the bottom of the sample at the given frequency range. The probe placed on the top of the sample is controlled by the near-field scanning system and conveys the measured results to the network analyzer.

References

[1] Z. Zhang, Q. Wei, Y. Cheng, T. Zhang, D. Wu, X. Liu, *Phys. Rev. Lett.* **2017**, 118, 084303.

[2] C. He, X. Ni, H. Ge, X.-C. Sun, Y.-B. Chen, M.-H. Lu, X.-P. Liu, Y.-F. Chen, *Nat. Phys.* **2016**, 12, 1124.

[3] W. A. Benalcazar, T. Li, T. L. Hughes, *Phys. Rev. B* **2019**, 99, 245151.

[4] J. C. Y. Teo, T. L. Hughes, *Phys. Rev. Lett.* **2013**, 111, 047006.

[5] W. A. Benalcazar, J. C. Y. Teo, T. L. Hughes, *Phys. Rev. B* **2014**, 89, 224503.

[6] X. Zhang, H.-X. Wang, Z.-K. Lin, Y. Tian, B. Xie, M.-H. Lu, Y.-F. Chen, J.-H. Jiang, *Nat. Phys.* **2019**, 15, 585.

[7] L. H. Wu, X. Hu, *Phys. Rev. Lett.* **2015**, 114, 223901.

[8] L. Zhang, J. Ding, H. Zheng, S. An, H. Lin, B. Zheng, Q. Du, G. Yin, J. Michon, Y. Zhang, Z. Fang, M. Y. Shalaginov, L. Deng, T. Gu, H. Zhang, J. Hu, *Nat. Commun.* **2018**, 9, 1481.
